# Rare Case of Osteochondroma on the Dorsal Aspect of the Scapula

**DOI:** 10.7759/cureus.17051

**Published:** 2021-08-10

**Authors:** Osama Shahid, Mubasshar Shahid, Likhita Shaik, Moavaz Masud, Shaheryar Ranjha

**Affiliations:** 1 Internal Medicine, CMH Lahore Medical College and Institute of Dentistry, Lahore, PAK; 2 Internal Medicine, Ashwini Rural Medical College, Hospital and Research Centre, Solapur, IND; 3 Cardiovascular Disease, Mayo Clinic, Rochester, USA; 4 Medicine, CMH Lahore Medical College and Institute of Dentistry, Lahore, PAK; 5 Internal Medicine, Akhtar Saeed Medical and Dental College, Lahore, PAK

**Keywords:** osteochondroma, benign bone neoplasm, dorsal scapula, exostosis, intramembranous ossification, scapula

## Abstract

Osteochondroma, often referred to as exostosis, is the most common benign bone tumor characterized by a bony protuberance surrounded by a cartilaginous surface. Most osteochondromas are found on the metaphysis of long bones, with the dorsal aspect of the scapula being a rare site of occurrence for an exostosis. Radiographic imaging, preferably through MRI or CT, assists in the identification of benign growth; however, a definitive diagnosis requires a biopsy. Open surgical resection and arthroscopic excision are the definitive treatment modalities of the nidus. Postoperative care requires immobilization of the limb for two months, with at least four months being the appropriate timeline for complete recovery.

## Introduction

Osteochondroma is an exostosis defined as a benign bony outgrowth with a cortical layer continuously projecting from the underlying bone, filled with cancellous bone and covered by cartilaginous tissue. Osteochondroma accounts for 35-46% of all benign bone neoplasms. The incidence of osteochondroma under the age of 30 is higher in men compared to women [1,2]. The outgrowth is usually found on the metaphyseal region of long bones such as the tibia, humerus, and distal femur, accounting for 90% of all exostoses [2,3]. Flat bones, such as the scapula, account for 3-4.6% of all cases of osteochondroma, with 14.4% of scapular tumors being diagnosed as osteochondroma [4,5]. The dorsal aspect of the scapula is a rare site of occurrence compared to the ventral aspect of the scapula, which comprises most cases of scapular exostoses [6,7].

## Case presentation

A healthy 23-year-old male presented to the CMH Orthopedic Outpatient Clinic with a complaint of a painless protrusion on the left side of his upper back for the past nine years. Although the protuberance was first noticed by the patient’s mother at age 13, no management was instituted till now. The mass slowly grew till the age of 18, after which its growth was arrested. At presentation, the patient reported a reduced range of motion of the left arm on full abduction. Otherwise, the patient’s history was unremarkable. He reported that his main reasons for visiting were cosmesis and reduced range of motion.

**Figure 1:**
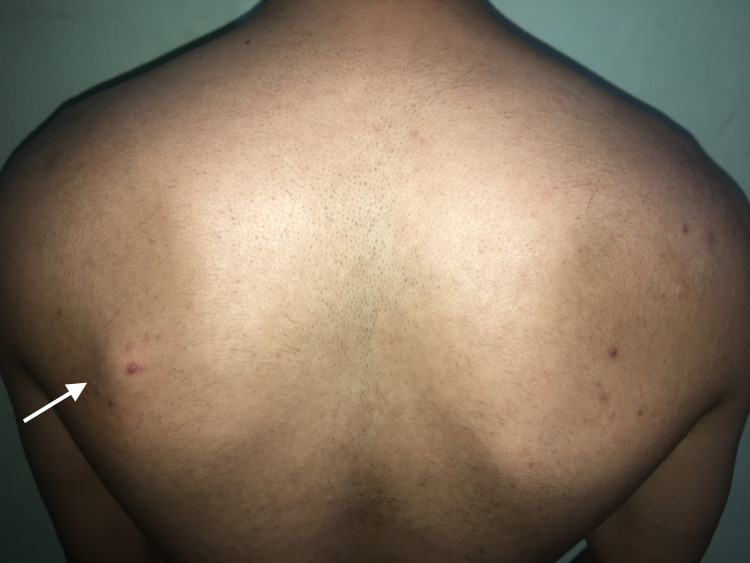
Arrow showing a hard, palpable mass on the dorsomedial aspect of the left scapula.

**Figure 2:**
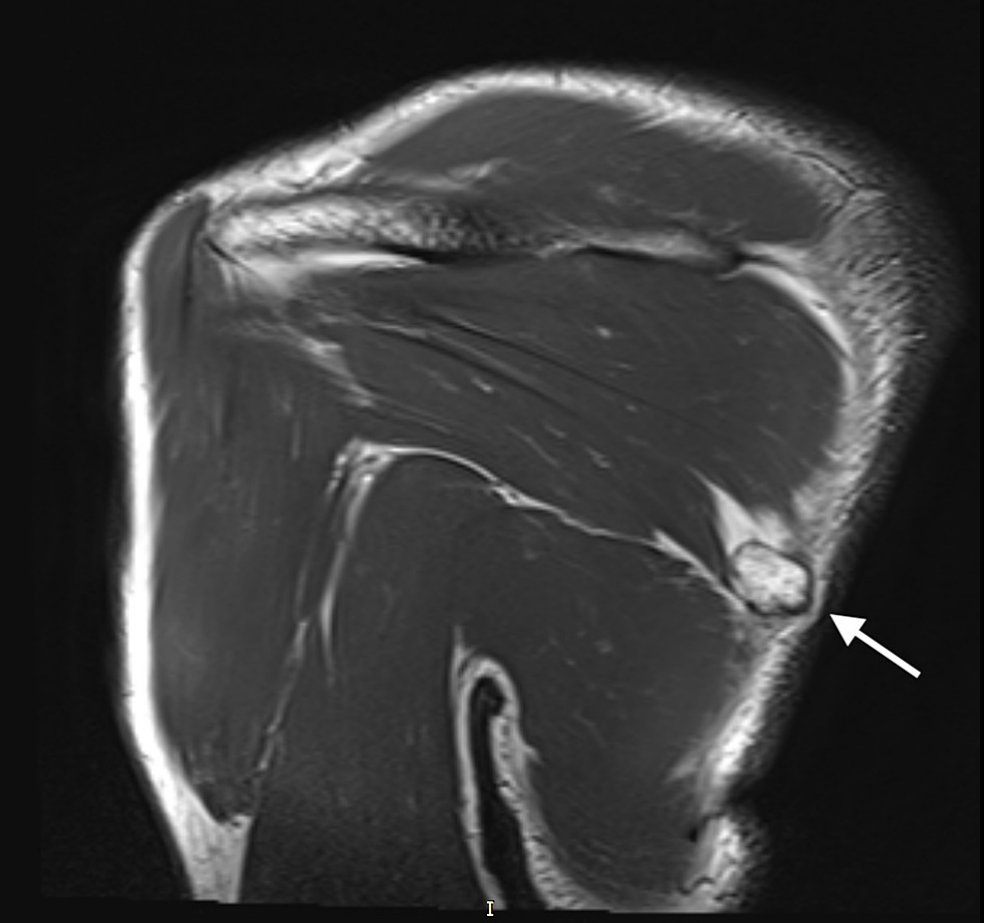
MRI coronal view showing a hyperintense lesion on the T2-weighted image measuring 1.58 × 1.80 × 1.54 cm (AP × TR × CC) at their maximum lengths on the dorsomedial aspect of the left scapula. MRI: magnetic resonance imaging; AP: anteroposterior; TR: transverse; CC: craniocaudal

**Figure 3:**
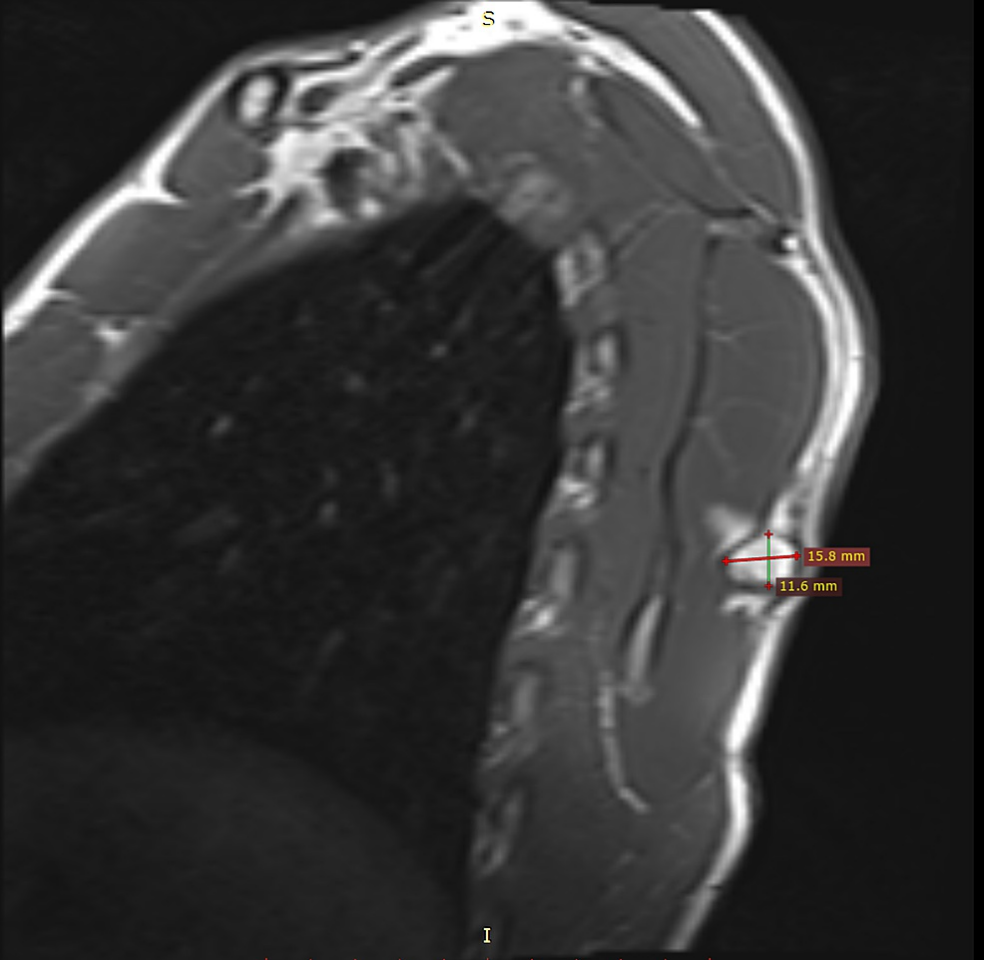
MRI sagittal view with the exostosis on the dorsal aspect of the left scapula. MRI: magnetic resonance imaging

On physical examination, a uniform, rounded, protuberant, and sessile nidus was seen and palpated in the medial region of the dorsal aspect of the left scapular blade (Figure 1). Although there was no tenderness to palpation, the range of motion of the left arm was restricted to 160 degrees on abduction. There was no evidence of winging of the scapula, even though the symmetry of the upper body upon full abduction of the left arm showed slight unevenness. Neurovascular structures were intact on examination of both the upper limbs. MRI revealed a hyperintense lesion on the T2-weighted images measuring 1.58 cm × 1.80 cm × 1.54 cm (anteroposterior × transverse × craniocaudal) at their maximum lengths on the dorsomedial aspect of the left scapula (Figures 2, 3). The imaging was consistent with the findings of a benign bone tumor. Although benign bone tumors such as enchondroma, fibroma, chondroblastoma, osteoid osteoma, osteoblastoma, and periosteal chondroma were part of the differential diagnoses, the radiographic appearance of a solitary osteochondroma was pathognomonic. The imaging showed protuberance of cortical and medullary bone from the underlying bone which confirmed the diagnosis.

The patient’s surgery has been scheduled for early 2022 for cosmetic reasons and to prevent future mechanical complaints. He is scheduled to be operated under general anesthesia in the prone position. An open incision will be made along the border of the scapula where the protrusion is most prominent. Muscle traction will be applied, after which extraperiosteal resection of the lesion will be attempted. Along with postoperative care, steroids will be injected into the operative wound to prevent keloid formation. The patient will be followed up for up to one year to monitor for recurrence of the swelling.

## Discussion

Osteochondroma is the most commonly occurring benign bone tumor accounting for 8% of all bone tumors. It occurs because of herniated epiphyseal cartilage into the bone through a periosteal defect during the ossification process. Due to this mechanism of growth involving the cartilage, bones that develop through intramembranous ossification have a lower predilection for this tumor. Sessile variants occur more frequently than pedunculated ones. The most common sites of occurrence include long bones (90%) such as the femur, tibia, and humerus [8-10]. Our patient presented with a dorsal surface scapular osteochondroma which is an unusual location considering an incidence rate of 3-4.5% compared to the incidence in long bones. The location of scapular tumors is divided into two zones [11]. Overall, 14.4% of scapular tumors are osteochondromas, and the ventral surface is more commonly involved compared to the dorsal surface. Osteochondroma of the scapula presents with chronic pain, cosmetic deformities, pain on motion, crepitus, decreased range of motion, weakness of the shoulder girdle muscles, and other compressive effects on the surrounding anatomical structures [8].

Plain radiographs such as X-rays are sufficient for the diagnosis of osteochondroma in long bones; however, more specialized modalities such as MRI are used to diagnose osteochondroma on flat bones. If not treated appropriately, complications such as pseudo-winging, restricted movement of the shoulder due to fibrosis, snapping, and bursa formation may occur [12,13]. As osteochondroma follows the course of bone growth until the closure of the physis, a small-sized osteochondroma in age groups ranging from pediatric to young adults can be carefully monitored and followed expectantly. Conservative management techniques include immobilization, physiotherapy, anti-inflammatory medications, and local anesthetic injections. Osteochondromas that cannot be managed conservatively require open or arthroscopic excision of the tumor. However, with larger tumors, it may be impossible to preserve the scapula by performing partial or complete scapulectomy. Though malignant transformation is rare in a solitary osteochondroma, multiple osteochondromas and sudden growth after the third decade of life must raise suspicion of its malignant transformation into osteosarcoma, which necessitates appropriate management [9,11]. This change is evidenced by the measurement of cartilage thickness of more than 2 cm on a CT scan or MRI. Lobulations, fibrous banding, myxoid changes, and gadolinium enhancement are other signs of malignancy. Our case is an atypical presentation of osteochondroma in a young adult patient due to its location. Clinicians must monitor the changing features of an osteochondroma to guide further management.

## Conclusions

Osteochondroma involving rare locations such as the scapula can be challenging to diagnose for clinicians. Fortunately, our case presented as a bony nidus on the dorsal aspect of the scapula with a recognizable protuberance on gross examination. We recommend including osteochondroma in the differential diagnosis of swelling or pain involving the bony structures of the scapular region.
